# Prevalence and therapeutic impact of adverse life event reexperiencing under ceremonial ayahuasca

**DOI:** 10.1038/s41598-023-36184-3

**Published:** 2023-06-09

**Authors:** Brandon Weiss, Aleksandra Wingert, David Erritzoe, W. Keith Campbell

**Affiliations:** 1 Division of Psychiatry, Imperial College London, London, UK; 2 Present Address: University of Georgia, Athens, GA USA

**Keywords:** Health care, Therapeutics

## Abstract

The present study examined the safety and efficacy of the ceremonial use of ayahuasca in relation to reports of heightened life event reexperiencing under psychedelics. The study examined (1) the prevalence of specific types of adverse life event reexperiencing, (2) characteristics predictive of reexperiencing, (3) the psychological character of reexperiencing, and (4) the impact of reexperiencing on mental health. Participants were recruited from three ayahuasca healing and spiritual centers in South and Central America (N = 33 military veterans, 306 non-veterans) using self-report data at three timepoints (Pre-retreat, Post-retreat, 3-months post-retreat). Reexperiencing adverse life events under ayahuasca was common, with women showing particularly high probability of reexperiencing sexual assault, veterans reexperiencing combat-related trauma, and individuals with a self-reported lifetime diagnosis of post-traumatic stress disorder exhibiting a substantively higher prevalence of reexperiencing. Reexperiencing was associated with states of cognitive reappraisal, psychological flexibility, and discomfort during ceremonies, and participants who reexperienced adverse life events exhibited greater reductions in trait neuroticism following their ceremonies. Clinical implications of these results for the application of psychedelics to mood and stress disorders are discussed.

## Introduction

As psychedelic therapies become increasingly accepted as tools for mental health care, many properties of psychedelic experience pertinent to clinical efficacy and safety remain unknown. Paramount among these is the degree to which moderate-to-large doses of psychedelics bring to the surface autobiographical memories of adverse life events and the impact of these memories on long-term mental health outcomes. These properties of psychedelic experience are important to explore as they can inform psychedelic therapy’s suitability for trauma-focused application as well as the need for consent and safeguards surrounding psychedelic-induced trauma re-exposure. Ceremonial ayahuasca and other psychedelic therapies have been proposed by a number of scholars to hold potential for treating post-traumatic stress disorder (PTSD)^1,2^, with clinical trials examining psilocybin-assisted therapy for PTSD currently underway, and early-stage biotechnology companies joining the exploratory effort. Meanwhile, ayahuasca shamans and ayahuasca healing centers in South America heavily focus on healing trauma, and have become increasingly popular among veterans seeking trauma-focused solutions. However, some clinical scientists have voiced concerns regarding the safety of psychedelic therapies for populations with a trauma history and PTSD, noting the potential for traumatic re-exposure of an uncontrolled and challenging nature^3^. These concerns are well-considered in view of known reports of unpleasant emotional reactions^4^ and “bad trips”^5^; as well as clinical scientists’ point of reference in therapies such as *Prolonged Exposure Therapy*^6^, wherein exposure is graduated, controlled, and can be stopped at any time.

Despite these ongoing questions about efficacy and safety, very little empirical data exists to inform them. The purpose of this study is accordingly to investigate the prevalence, proximal etiology (i.e., predictors of recollection), character, and impact of adverse life event reexperiencing during the ceremonial use of ayahuasca and psychedelic experience more broadly. We undertake this investigation in a large sample of adult ayahuasca retreat-goers and a smaller sample of veterans, many of whom have experienced combat-related trauma, served as special operations forces, and possessed a previous diagnosis of PTSD. We believe this empirical study is the first to systematically examine rates of re-exposure to adverse life events under psychedelic drugs, and its consequences for mental health.

### Importance of psychedelic therapies in veteran mental health

Exploring these questions in the combat-veteran population is particularly important for two reasons. First, there is concern within the veteran community regarding the adequacy of current psychotherapeutic and pharmacotherapeutic treatments for PTSD. Veterans are increasingly utilizing non-profits such as Heroic Hearts Project, Veterans Exploring Treatment Solutions, and Special Operations Care Fund to pursue treatment alternatives to Veteran Affairs-sponsored mental health services, such as repetitive transcranial magnetic stimulation therapy, hormone replacement, and various psychedelic treatments. Preliminary research on the efficacy of these treatments has been promising^7^, and MDMA-assisted therapy has demonstrated key strengths relative to gold-standard trauma-focused therapies, chiefly greater tolerance and lower dropout^8^.

Second, many veterans, particularly those with a special operations forces background, present with a symptom profile that deviates from the traditional Diagnostic and Statistical Manual of Mental Disorders (DSM)^9^ PTSD category^10^, raising the possibility that alternative therapies may be better suited. For example, “Operator Syndrome”^10^ describes a complex array of problems that are thought to stem from operators’ specialized combat training as well as frequent blast-wave exposure. Problems include endocrine dysfunction, sleep disturbance, headaches, memory, concentration, and other cognitive impairments, hypervigilance, interpersonal dysfunction, and challenges in adapting to civilian life. Symptoms of grief, survivor’s guilt, and a sense of foreshortened future are also more common in this population, stemming from their familiarity with mortality and loss, whereas Criteria B and C symptoms involving intrusive thoughts and fear/avoidance are less frequently reported^10^.

### Psychedelic reexperiencing of autobiographical memories

A number of quantitative and qualitative sources link psychedelic compounds, including ayahuasca, to increased autobiographical recollection of previous adverse life events (see Healy^11^ for review). Direct examinations of the effect of psychedelic compounds on autobiographical recollection however remain limited and mixed. Carhart-Harris et al.^12^ observed fMRI hyperactivation in visual and sensory brain areas and increased subjective reports of vivid and visual recollection in relation to autobiographical memory cues among healthy participants under psilocybin versus placebo. These findings reflect the possibility of more vivid recollections, but not necessarily more prevalent recollections under psychedelics. Speth et al.^13^ reported *reduced* mental time travel to the past under lysergic acid (LSD) in a small sample of healthy volunteers, suggesting a lower prevalence of autobiographical memories.

Examinations of clinical trial and naturalistic survey data have shown preliminary evidence for increased autobiographical reexperiencing under psychedelics as well as its therapeutic value. Three clinical trials examining the efficacy of psilocybin-assisted therapy for anxiety, depression, and cigarette addiction^14–16^ have reported patient reexperiences of childhood sexual assault^17,18^ and previous physical and emotional abuse in infancy and childhood^19,20^ during dosing sessions. In addition, five naturalistic studies examining therapeutic mechanisms specific to ayahuasca have similarly shown reports of autobiographical memory reexperiencing^2,21–23^, with some asserting ayahuasca-mediated recollection of suppressed or forgotten memories^24^. In general, the foregoing reports ascribe therapeutic value to autobiographical reexperiencing, with some patients suggesting that engagement with these memories catalysed healing^17^, and others attributing the clinical trial’s therapeutic efficacy primarily to productive engagement with previous trauma^19^. These reports have prompted some scholars and practitioners to point to reexperiencing and associated biological processes as potential mechanisms underlying action on PTSD and other disorders bearing a trauma-related etiology^1,25^.

Notwithstanding the importance of the foregoing findings, a number of gaps remain. First, these observations were generally obtained from small samples in which adverse life event recollections were not systematically recorded, limiting their ability to yield precise prevalence rates. Second, little is known about how different trauma types (e.g., sexual assault, accident) may be differentially associated with the probability of psychedelic reexperiencing. Furthermore, little is known about whether populations that disproportionately experience certain trauma types (e.g., sexual assault in women^26^, disaster, harming others, loss in combat-veterans^27^) are predisposed to a higher probability of reexperiencing such events.

### Proposed mechanisms underlying trauma-focused psychedelic therapy

Multiple theories have been put forth accounting for psychedelics’ therapeutic action on PTSD at psychoanalytic, cognitive, and biological levels of analysis. Psychoanalytic theories hold that ayahuasca diminishes defense mechanisms that typically impede the emergence of challenging psychological material^21,24^. Consistent with emotional processing theory^28^, psychedelic experience may also assist in activating and modifying trauma memory structures through exposure to avoided stimuli (i.e., memory content) and extinction learning^25^. Similar to the imaginal exposure component of *Prolonged Exposure Therapy*, engagement with trauma memories under psychedelics may accompany^1^ reorganizing the memory, ^2^ re-examining negative appraisal of conduct during the remembered episode, ^3^ distinguishing between remembering the trauma and experiencing the trauma, and ^4^ conveying understanding that the trauma can be remembered without resulting in harm^29,30^). Wolff et al.^25^ propose that psychedelics may be particularly effective in revising trauma memory structures through producing sustained, involuntary exposure. Sustained exposure may also be consistent with deficits in cognitive control observed under psychedelics^31,32^, whereby it is plausible that attentional shifts away from trauma memories are impaired. As a note, exposure-supported extinction learning is the dominant explanation for the action of MDMA-assisted therapy for PTSD^33^, and may also apply to psychedelic therapies.

Other scientists have observed the role of psychological flexibility in psychedelic-mediated healing^34–36^. Psychological flexibility is defined as the ability to flexibly alternate between and manage challenging thoughts, emotions, or mental sets in the service of carrying out valued actions^37^. Given that psychological *inflexibility* has been implicated as a maintaining factor in multiple mental health disorders including PTSD^38^, it is possible that enhanced flexibility under ayahuasca may accompany therapeutic processes, e.g., cognitive processing of previous trauma and trauma-related beliefs^39^, fear extinction.

Finally, the entropic brain hypothesis^40^ and free energy principle^41^ have also been invoked^25^ to account for the emergence of trauma memories under psychedelics as well as for their potentially therapeutic action^25^. Combined in the *relaxed beliefs under psychedelics theory* (REBUS)^42^, the theory conceptualizes the brain as an inference-making machine that learns about the world through making predictions, avoiding uncertainty, and updating in response to new (or deviant) external percepts. Through producing entropy (i.e., the degree to which neural activation patterns cannot be predicted by prior states of activation, disorder) in the higher-level (transmodal) control areas of the brain, psychedelics are thought to relax the rigidity of the predictions, which can themselves exert a constraining influence over mental experience. This is theorized to allow an opening of the mind to new sensory (and generally mental) experience, which may revise certain higher-order beliefs about oneself and one’s world. In a therapeutic context, such potential for revising beliefs could conceivably guide reappraisal of rigid maladaptive beliefs underlying mood and stress disorders. For example, a patient with sexual assault history may hold rigid negative beliefs about their blameworthiness and their surroundings’ high risk of harm. Under psychedelics, the weighting of these beliefs may be relaxed, allowing the person to explore alternative perspectives.

### Present study

The present study investigated the prevalence, proximal etiology (i.e., predictors of traumatic recollection), character, and impact of adverse life event reexperiencing in relation to the ceremonial use of ayahuasca in a sample of 306 non-veterans and 33 veteran participants. Two aims were primary. First, we aimed to examine the prevalence of adverse life event experience before ayahuasca use and reexperience during use, with focus on multiple types of adverse life events, ranging in severity. Differences between populations (females vs males, veterans vs non-veterans) were examined to identify unique group-level risks and treatment opportunities. Second, we aimed to prospectively examine the therapeutic effect of reexperiencing on mental health, using Five-Factor model (FFM)^43^ of personality Neuroticism scores. We examined Neuroticism given its versatility as a measure of normal-range negative affectivity and psychopathological-range internalizing^44^, and its no less dynamic responsiveness to therapy and life experience^45–47^. Notably, Neuroticism bears high overlap with the shared variance of internalizing disorders^48^, making it ideal as a measure of this principal component of psychopathology.

Secondary aims were to examine predictors of adverse life event reexperiencing to guide enhanced reliability of acute psychedelic effects. We furthermore examined which acute experiences (e.g., mystical-type experience, cognitive reappraisal, discomfort) may covary with reexperiencing, which may be suggestive of either the proximal precursors to reexperiencing or the cognitive states involved in reexperiencing.

## Materials and methods

### Study procedure, recruitment, and participants

Three-hundred-fifty-one participants were recruited from three ayahuasca retreat centers across South and Central America: Arkana Spiritual Center (Requena, Loreto, Peru), Soltara Healing Center (Gulf of Nicoya, Costa Rica), and La Medicina (Cordilliera Escalera mountain range, Peru) with the assistance of center intake directors and the Heroic Hearts Project, which sponsors alternative mental healthcare options for veterans.

Participants were emailed two weeks before the date of their ayahuasca retreat with an invitation to enroll. In the recruitment consent form, participants were informed that some of the study questionnaires would include questions relating to previous “traumatic experiences” and to “their experiences during the use of ayahuasca in ritual context.” They were informed that some of the questions may be sensitive in nature, and that they had the ability to opt out of answering any questions they did not feel comfortable answering for any reason. Inclusion criteria included being above 18 years of age.

Participants were informed that they would be compensated with entry into a raffle for a week-long retreat (valued at $1580.00). Further monetary incentives of $20.00 and $30.00 were additionally offered with the second and third survey reminders, respectively, to promote compliance and reduce attrition.

On average, study surveys were administered nine days before the retreat start date (baseline), five days after the retreat end date (post), and three months following the retreat end date (~ ± 114 days after retreat end date; follow-up). Data was collected using online surveys supported by the Qualtrics web platform. For full details on study design and procedure, see Weiss and colleagues^49^.

All participants provided online informed consent in accordance with the Common Rule and the Declaration of Helsinki. All procedures were performed in accordance with the relevant guidelines and regulations of the University of Georgia Institutional Review Board.

### Retreat program

The retreat program varied across centers. In general, on the second morning, participants ingested a vomitivo (e.g., Kambo, lemongrass) to induce vomiting. Facilitators subsequently provided an introduction to ayahuasca ceremonial practices and answered questions. Participants typically had occasion to meet individually with shaman ayahuasqueros (in the Shipibo lineage) and facilitators to inform them about their purpose for conducting ceremony and to ask any personal questions. Facilitators requested that participants formulate intentions to bring into ayahuasca ceremony, but these were not formally communicated to ayahuasqueros or facilitators. Ayahuasqueros and facilitators did not engage in any consistent process of guiding participants to remember previous adverse life events, although individual participants may have referenced previous life events in their individual session.

Before ayahuasca ceremonies, participants had flower baths, in which ayahuasqueros poured water with fragrant floral plants over the participants. This ritual is considered to provide protection from certain spirits during ceremonies. During ayahuasca ceremonies, participant group-members drank ayahuasca one by one, contemplating their intentions while drinking. Participants were arranged on individual mats within a spacious maloca while two curanderos typically of mixed gender sang icaro prayers. At some centers, the ayahuasqueros sat in front of participants at opposite sides of the maloca, addressing their songs to one individual at a time before transitioning to the next as they circumambulated the circular perimeter of the maloca. At other centers, the ayahuasqueros sat in one place throughout the ceremony, and participants were brought up to them to receive their icaro. The icaro is a prayer regarded to have originated from previously “dieted” traditional plants/spirits. In the Shipibo ayahuasqueros’ worldview, the icaro opens up “portals” that guide positive spirits (operating as “muses” or “doctors”) to remove “dirty” or “calcified” energy from ceremony participants. In the darkness of the maloca, the ayahuasqueros were typically identified by a faint silhouette and the glowing red embers of their mapacho (tobacco grown in the Peruvian amazon) cigar. In the maloca space, it was typically possible to overhear the utterances of other participants. During ceremony, participants were typically able to request additional portions of ayahuasca from facilitators, as well as to smoke mupacho cigars. On days following ceremony nights, group shares typically took place, in which participants communicated their unique experiences and insights with the larger group.

On average, participants drank ayahuasca in 4.44 (SD = 2.20) ceremonies, over 1.38 (SD = 0.61) weeks, and drank one full glass of ayahuasca per ceremony.

### Measures

#### Adverse life events experience (ALE)

The Life event checklist for DSM-5 (LEC-5)^50^ is a self-report inventory that measures 17 types of discrete experiences of adversity (e.g., natural disaster, fire or explosion, physical assault, sexual assault). To reduce the burden on participants, the LEC-5 was adapted to contain nine types of adverse life events (ALE) including (1) natural disaster, fire, or explosion, (2) transportation or serious accident at home, work, or during a recreational activity, (3) physical assault, assault with a weapon, (4) sexual assault, (5) Other unwanted or uncomfortable sexual experience, (6) life-threatening illness or injury, (7) sudden death of another, (8) serious injury, harm, or death [one] caused someone else, and (9) any other very stressful event or experience. See Fig. S1 to observe the LEC-5 and adapted study questionnaire. During baseline assessment, participants were asked whether they had experienced each of these events. Following their ceremonies, participants were asked whether they reexperienced any of these events during their ceremonies. Participants who endorsed reexperiencing an ALE were subsequently asked the intensity of their reexperience using a 5-point Likert-scale (1 = Not intense, 5 = Very intense).

Six additional variables were computed from LEC-5 data. First, a binary *Any adverse life event* variable was computed that indexed whether or not participants endorsed one or more events previously. A reexperiencing-related *Any adverse life events* variable was additionally computed related to events reexperienced during ceremony. Second, a more severe version of the *Any adverse life events* variable (*Any severe adverse life events*) was computed that omitted stressful experiences and sexually uncomfortable experience. A reexperiencing-related *Any severe adverse life events* variable was computed related to events reexperienced during ceremony. Third, an *ALE Index* variable was computed that summed the number of discrete events that participants reported. A *Reexperiencing Index* variable was also computed.

#### Five factor model personality domains

A 120-item set of the International Personality Item Pool (IPIP-NEO-120)^51^ was used to index self-reported personality traits. The IPIP-NEO-120 consists of five 24-item FFM domain subscales (Neuroticism, Extraversion, Openness, Agreeableness, Conscientiousness). Items used a 5-point Likert-type scale (1 = Strongly disagree, 5 = Strongly agree). The IPIP-NEO-120 has demonstrated good reliability and construct validity when compared to the Revised NEO Personality Inventory^43,51^. FFM domains have shown adequate test–retest reliability across an average interval of four weeks (rs > 0.77)^52^. Longitudinal measurement invariance has also been supported at the metric and scalar level in large samples^53,54^. Neuroticism was used as an index of mental health at baseline, post, and 3-month follow-up, whereas the other traits were used as predictors of reexperiencing. Internal consistency for Neuroticism ranged from 0.92 (3-month follow-up) to 0.93 (baseline); and for the other domains ranged from 0.79 (Agreeableness) to 0.92 (Extraversion).

#### Measures of acute experience

##### Mystical experience

The Mystical Experience Questionnaire (MEQ-30)^55^ is a 30-item scale used to assess mystical aspects of participants’ experiences during their ayahuasca ceremonies. For the purpose of this study, the Mystical subscale (15-item; e.g. “Experience of the fusion of your self into a larger whole”) was used. Items used a 6-point Likert-type scale (1 = None, 6 = Extreme (more than any other time in my life and stronger than 5)) and asked participants to consider the degree to which they had experienced mystical phenomena at any time during the ceremonies. One of the items from the Mystical subscale (item 30) was excluded due to administrator error, but internal consistency remained strong (α = 0.95).

##### Ayahuasca experience

The Ayahuasca Experience Inventory^56^ was developed to measure thoughts, feelings, behaviours, and attitudes that arise within ayahuasca ceremonies. The AEI consists of three factors: Clarity (32 items; α = 0.97) captures clarity, peace, self-connection, and self-esteem; Reappraisal (30 items; α = 0.96) captures cognitive reappraisal of negative beliefs about self/others as well as the meaning of challenging life experiences; and Discomfort (15 items; α = 0.94) captures unpleasant feelings of torment, discomfort, and isolation that seemed unending. Items asked participants to consider the degree to which they had experienced phenomena at any time during the ceremony. Items were measured using a 6-point Likert scale (1 = None, 6 = Extreme (more than any other time in my life and stronger than 4)). The AEI Reappraisal scale is a novel measure that was piloted in the primary study^49^. As the scale is not yet published, more explanation of its psychometric properties is provided in Supplementary Materials II).

#### Lifetime diagnosis of post-traumatic stress disorder

During baseline assessment, participants were asked to share, with open-ended responses, any mental health diagnoses they had previously received. These responses were then coded and a categorical, binary variable was computed indexing the presence or absence of participants’ previous diagnosis of PTSD.

#### Qualitative intention variables

At post-retreat assessment, participants were asked to describe their pre-ceremony intentions open-endedly (“What intentions did you bring into your ceremonies?”). Responses were then coded for whether they contained intentions related to either resolving stress surrounding previous ALEs (*Trauma Healing Intention*) or more general mental health-related healing (*Healing Intention*). The first and second authors both performed this coding, and deliberated in cases of disagreement to reach consensus. The *Trauma Healing Intention* and *Healing Intention* variables were categorical and binary, with each participant scoring either 0 (absent) or 1 (present).

#### Validity criteria

Two 8-item validity scales from the Elemental Psychopathy Assessment^57^ were used to detect invalid responding on measures of personality. These scales were the Infrequency scale and the Unlikely Virtue scale. In line with guidelines^57^, participants who endorsed more than three Infrequency scale items and more than two Unlikely Virtue scale items were eliminated.

#### Ceremony characteristics

Participants were asked about characteristics of their retreat experience including the number of ceremonies in which they consumed ayahuasca, retreat length, average dosage of ayahuasca, and experiences with other plant medicines (e.g., huachuma, sapo). Dosage was computed as the approximate ayahuasca quantity consumed across ceremonies in terms of glasses (e.g., one and one-half glasses). Of note, glass size was not standardized and varied within and across retreat centers. Complete data for dosage and frequency of ceremonies were available for 276 participants.

### Analytic plan

Five primary sets of analyses were planned. The statistical significance threshold was set at *p* < 0.05, and the Benjamini and Hochberg^58^ False Discovery Rate (FDR) adjustment was applied to correct for multiple comparisons, using the Sgof R package^59^. All data met assumptions of normality according to guidelines indicated by^60,61^.

The first set of analyses counted the number of veterans and non-veterans who reported previous adverse life events and reexperiences of previous adverse life events during ceremony. The second set of analyses tested differences in the prevalence of ALEs and reexperiencing between subsamples (veterans vs non-veterans; PTSD diagnosis vs no PTSD diagnosis) and sexes. The third set of analyses calculated correlations between acute factors and reexperiencing.

In the fourth set of analyses, linear mixed effects (LME) models were conducted using the ‘lme4’^62^ package in R software to examine the moderation-based effect of previous ALE experience and ALE reexperiencing on change in *Neuroticism*. *Neuroticism* scores were regressed onto the interaction between *Timepoint* (a categorical variable with levels baseline, post, and follow-up) and each moderator, separately, with a random effect of intercept specified. As such, these models were used to test whether ALE experience or reexperience scores significantly moderated estimated changes in *Neuroticism*, i.e., the effect of *Timepoint* on *Neuroticism*. Because all timepoints were included in the model, results were indicative of the degree of moderation of change between baseline and post, and between baseline and follow-up. Given a positive moderation-based effect of age on change in *Neuroticism*, *Age* was included as a covariate. Accordingly, the mathematical model and R code are presented below:where Yij = level of *Neuroticism* for person j at timepoint (categorical variable with levels of baseline, post, follow-up). For these analyses, we generated unstandardized (B) coefficients for the interaction term, representing the effect of a one-unit increase in the moderator on the magnitude of the relationship between Timepoint and Neuroticism (i.e., difference between two timepoints in Neuroticism scores).

**Table:** 

Mathematical model	Level 1: Yij = β0j + β1j(Timepoint)j + β2j(Age) + rij
	Level 2: β0j = γ00 + γ01(Moderator)j + u0j (random intercept)
	Level 2: β1j = γ10 + γ11(Moderator)j
R formula	lmer(Y ~ Timepoint*Moderator + Age (1 | participant) , data = data frame)

Five supplementary sets of analysis were also undertaken. No adjustment for multiple comparisons was used for these analyses, attending inflated Type I error. The first set of analyses examined whether change in *Neuroticism* was significantly greater in the veteran versus non-veteran sample (Supplementary Materials III). The first set of analyses calculated correlations between baseline characteristics (demographic characteristics, baseline traits, pre-ceremony intentions) and reexperiencing ALEs (Supplementary Materials IV). The third set of analyses identified open-ended responses in which participants reflected on how their ceremony experiences modified their relationship to previous ALEs (Supplementary Materials V).

### Ethical approval

This research was approved by the University of Georgia Institutional Review Board.

## Results

### Participant sample

Of 351 participants who were recruited, 2% (N = 6) did not meet the validity criteria (see Measures), and 2% (N = 6) did not report on previous adverse life events, leaving a final sample of 339 validly responding participants with adverse life event information. Three-hundred-thirteen participants of these responded at baseline and at least one subsequent time point (i.e., post, 3-months post-experience), and were thus eligible for prospective analyses. Sample size was originally determined on the basis of obtaining statistical power to detect a change in trait neuroticism of at least 0.20 standard deviations^49^.

#### Non-veteran sample

The final non-veteran sample consisted of 306 participants. Two-hundred-eighty-one participants of these responded at baseline and at least one subsequent time point, and were thus eligible for prospective analyses (mean age = 34.97 ± 10.03; ethnicity: 91% non-Hispanic White, 2% Black, 6% Asian American/Native Hawaiian or Other Pacific Islander, 7% Latinx, and 3% Indian). Because a larger number of participants reported on two timepoints versus all three, datasets were used to examine the change between baseline and post (N_full_ = 273; N_adverse life events endorsing_ = 229), and baseline and 3-month follow-up (N_full_ = 249; N_adverse life events endorsing_ = 207). The sample consisted of 183 participants who self-identified as males (N_adverse life events endorsing_ = 153) and 121 who self-identified as females (N_adverse life events endorsing_ = 106).

Approximately 19% of participants reported an annual income of less than $30,000, 29% reported annual income greater than $30,000 but less than $60,000, 15% reported greater than $60,000 but less than $90,000, and 31% greater than $90,000. Approximately 28% of participants reported completing some high school, 38% reported graduating high school, 16% reported completing some college, and 17% reported obtaining at least a bachelor’s degree. Approximately 11% of participants had self-reported diagnoses of major depression, 4% with PTSD, and 5% with anxiety disorder.

#### Veteran sample

The final veteran sample consisted of 33 participants. Thirty-two participants of these responded at baseline and at least one subsequent time point, and were thus eligible for prospective analyses (N = 30 at baseline and post, N = 30 at baseline and 3-month follow-up; mean age = 34.97, SD ± 10.03; ethnicity: 97% non-Hispanic White, 15% Latinx, and 6% Indian). The sample consisted of 30 participants who self-identified as males and three as self-identified females.

In total, 16 Army veterans (including two Green Berets, seven Rangers, one Special Air Serviceman, one Mortarman), 10 Marine veterans (including one Scout Sniper), three Navy veterans (including two SEALs), two Air Force veterans (including two Pararescuers), one Canadian Army special forces veteran, and one British Army Special Air Service veteran participated in retreats. Fifty-two-percent of the sample had a special operations forces background, 85% of veterans reported combat exposure, and 67% of veterans disclosed a lifetime PTSD diagnosis. Diagnosis was not directly assessed by study personnel.

Approximately 15% of participants reported an annual income of less than $30,000, 30% reported annual income greater than $30,000 but less than $60,000, 15% reported greater than $60,000 but less than $90,000, and 39% greater than $90,000. Approximately 33% of participants reported completing some high school, 36% reported graduating high school, 15% reported completing some college, and 15% reported obtaining at least a bachelor’s degree. Approximately 24% of participants had self-reported diagnoses of major depression, 67% post-traumatic stress disorder, and 12% anxiety disorder.

### Examining the prevalence of adverse life events and reexperiencing

#### Non-veteran sample

Of the 306 non-veteran participants, the majority (85%) reported previous experiences of ALEs, and 53% reported previous experiences of severe ALEs. The most prevalent ALE was stressful experience, reported by 54% of participants, whereas the most prevalent severe ALE was loss of a significant other, reported by 35% of participants. The most commonly reported ALEs ranged from 5% (perpetration) to 54% (stressful experience).

Of the 85% of non-veteran participants who endorsed a previous ALE, 42% reported reexperiencing a previous ALE during their ceremony experiences while under the acute effects of ayahuasca (mean intensity 3.94 ± 0.94). Of the 53% of participants who endorsed previous experiences of severe ALEs, 27% reported reexperiencing a severe ALE during ceremony (mean intensity 3.92 ± 1.07). The proportion of ALE-endorsing participants who reported reexperiencing at least one ALE ranged from 2% (disaster) to 39% (stressful experience). The proportion of severe ALE-endorsing participants who reported reexperiencing at least one severe ALE ranged from 2% (disaster) to 33% (sexual assault).

Notably, among severe ALE types, sexual assault carried the highest reexperiencing rate, such that among the 17% of participants endorsing previous sexual assault, 33% reported reexperiencing during ayahuasca ceremony (mean intensity = 3.86 ± 0.95). Sexually uncomfortable experiences were endorsed by 32% of participants, with 24% reexperiencing (mean intensity 3.38 ± 1.28).

**Figure 1:**
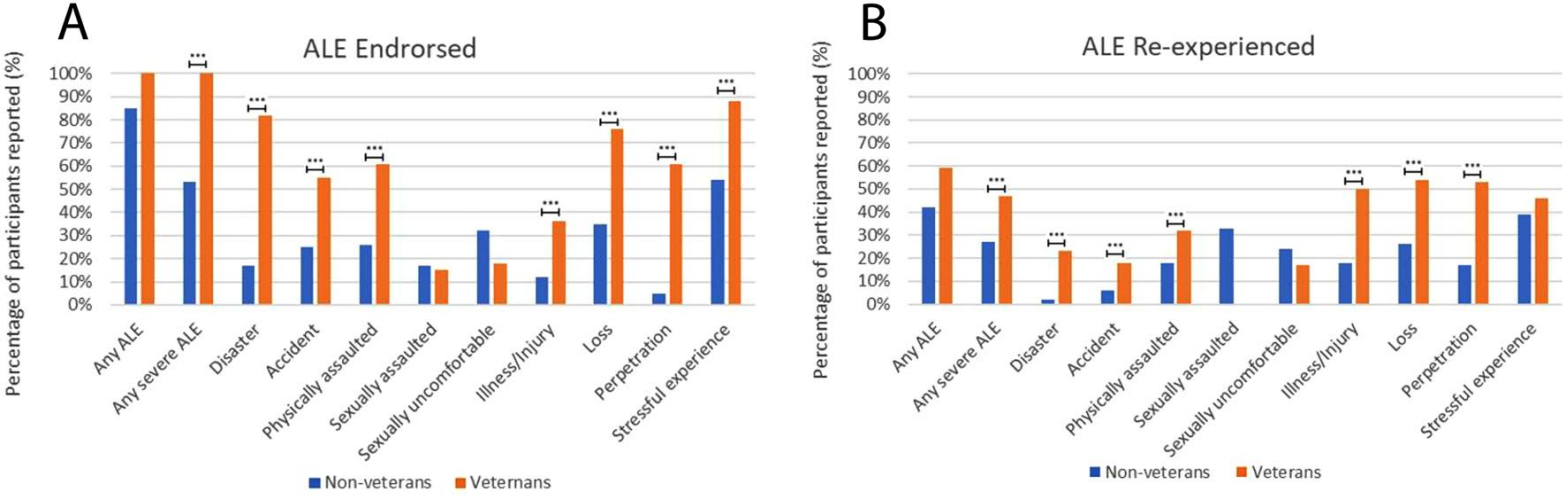
Percentage prevalence of ALE experiencing and ALE reexperiencing in military veterans (n = 33) and non-veterans (n = 306). Plot (**A**) shows differences between subgroups in the prevalence of ALE experience. Plot (**B**) shows differences in prevalence of ALE re-experience. Asterisks indicate statistically significant differences: *p < 0.05, **p < 0.01, ***p < 0.005.

Figure 1 and Table 1 illustrate the percentage of experiencing and reexperiencing associated with each ALE type and the mean intensity of participants’ recollections.

**Table 1:** Prevalence of adverse life event experience and adverse life event reexperience by sample.

	Adverse life event experienced			Adverse life event reexperienced			
	N	Participants reporting	Percentage	N	Participants reporting	Percentage	Intensity mean (SD)
Non-veteran sample							
Any adverse life event	306	260	85%	228	96	42%	3.94 (0.94)
Any severe adverse life event	306	162	53%^a^	139	38	27%^c^	3.92 (1.07)
Disaster	306	52	17%^a^	44	1	2%^c^	3.00 (NA)
Accident	306	76	25%^a^	67	4	6%^c^	3.00 (0.81)
Physically assaulted	306	79	26%^a^	72	13	18%^c^	4.00 (1.00)
Sexually assaulted	306	52	17%	42	14	33%	3.86 (0.95)
Sexually uncomfortable	306	99	32%	87	21	24%	3.38 (1.28)
Illness/Injury	306	38	12%^a^	34	6	18%^c^	5.00 (0.00)
Loss	306	108	35%^a^	93	24	26%^c^	3.71 (1.37)
Perpetration	306	16	5%^a^	12	2	17%^c^	4.50 (0.71)
Stressful experience	306	165	54%^a^	149	58	39%	4.25 (0.87)
Veteran sample							
Any adverse life event	33	33	100%	32	19	59%	3.79 (1.18)
Any severe adverse life event	33	33	100%^b^	32	15	47%^d^	3.95 (1.33)
Disaster	33	27	82%^b^	26	6	23%^d^	3.00 (1.41)
Accident	33	18	55%^b^	17	3	18%^d^	5.00 (0.00)
Physically assaulted	33	20	61%^b^	19	6	32%^d^	4.20 (1.79)
Sexually assaulted	33	5	15%	5	0	0%	NA
Sexually uncomfortable	33	6	18%	6	1	17%	NA
Illness/Injury	33	12	36%^b^	12	6	50%^d^	4.00 (1.55)
Loss	33	25	76%^b^	24	13	54%^d^	3.75 (1.42)
Perpetration	33	20	61%^b^	19	10	53%^d^	4.22 (1.56)
Stressful experience	33	29	88%^b^	28	13	46%	3.85 (1.28)

^a^ and ^b^ indicate statistically significant differences between non-veterans and veterans in ALE experience. ^c^ and ^d^ indicate significant differences between non-veterans and veterans in ALE reexperience.

#### Veteran sample

Of the 33 veteran participants, all reported experiencing severe ALEs prior to their participation in the study. The most commonly reported ALEs ranged from 15% (sexual assault) to 88% (stressful experience). Six of the nine ALE types were endorsed by more than 50% of veterans, including disaster, accident, physical assault, loss, perpetration, and stressful experience.

Fifty-nine percent of veterans reported reexperiencing an ALE during ceremonies (mean intensity 3.79 ± 1.18), and 47% reported reexperiencing a severe ALE (mean intensity 3.95 ± 1.33). The proportion of ALE-endorsing participants who reported reexperiencing at least one ALE ranged from 0% (sexual assault) to 54% (loss). Veterans reported reexperiencing illness/injury, loss, and perpetration at a proportion above 50%.

Figure 1 and Table 1 provide detailed results.

### Comparing the prevalence of adverse life events and reexperiencing between veterans and non-veterans

Veterans reported previous severe ALEs at significantly higher rates than non-veterans. Higher prevalence was observed for ALEs including disaster, accident, physical assault, illness/injury, loss, perpetration, and stressful experience (*p* < 0.005).

Veterans also exhibited a higher rate of reexperiencing severe ALEs (X^2^ = 12.09, *p* = 0.004), disaster (X^2^ = 37.34, *p* < 0.001), accident (X^2^ = 18.80, *p* < 0.001), physical assault (X^2^ = 13.73, *p* = 0.001), illness/injury (X^2^ = 20.32, *p* < 0.001), loss (X^2^ = 24.25, *p* < 0.001), and perpetration (X^2^ = 36.23, *p* < 0.001).

Figure 1, Table 1, and Table S2 present these results.

### Comparing the prevalence of adverse life events and reexperiencing between males and females

Male and female non-veteran participants were relatively similar in their reporting of previous ALE history. Of the 121 female participants, 88% endorsed at least one ALE, and 57% of women reported reexperiencing at least one severe ALE. Of the 183 male participants, 84% endorsed at least one ALE, and 52% endorsed at least one severe ALE. However, female participants exhibited a higher prevalence of previous ALEs involving sexual assault (32% vs 7% for males; X^2^ = 30.69, *p* < 0.001), and sexually uncomfortable experience (52% vs 20% for males; X^2^ = 33.35, *p* < 0.001).

Females generally reexperienced ALEs (57% females vs 32% males; X^2^ = 9.99, *p* = 0.008) at significantly higher rates than males. Contributing to this pattern, female participants reexperienced sexual assault at a significantly higher rate (41% females vs 10% males; X^2^ = 14.73, *p* = 0.001).

The veteran sample only included three individuals who identified as female; therefore, comparisons could not be validly undertaken. All three female participants reported previous sexual assault and sexually uncomfortable experience. However, reexperiencing rates could not be validly estimated as only one woman (33%) reported re-experiencing sexually uncomfortable behaviour, whereas two out of three did not volunteer a response.

**Figure 2:**
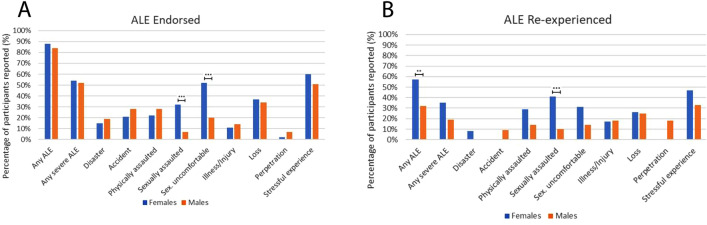
Percentage prevalence of ALE and ALE reexperiencing in non-veteran male (n = 183) and female (n = 121) participants. Plot (**A **) shows differences between subgroups in the prevalence of ALE experience. Plot (**B**) shows differences in prevalence of ALE re-experience. Asterisks indicate statistically significant differences: *p < 0.05, **p < 0.01, ***p < 0.005.

Figure 2, Table 2, and Tables S1 and S2 present these results.

**Table 2:** Prevalence of adverse life event experience and adverse life event reexperience by sex.

	Adverse life event experienced			Adverse life event reexperienced			
	N	Participants reporting	Percentage	N	Participants reporting	Percentage	Intensity mean (SD)
Female non-veteran sample							
Any ALE	121	106	88%	90	51	57%^c^	3.75 (1.04)
Any severe ALE	121	65	54%	53	22	42%	3.61 (1.16)
Disaster	121	18	15%	13	1	8%	3.00 (NA)
Accident	121	25	21%	20	0	0%	NA
Physically assaulted	121	27	22%	21	6	29%	3.50 (1.05)
Sexually assaulted	121	39	32%^a^	32	13	41%^c^	3.85 (0.99)
Sex. uncomfortable	121	63	52%^a^	52	16	31%	3.25 (1.13)
Illness/Injury	121	13	11%	12	2	17%	5.00 (0.00)
Loss	121	45	37%	39	10	26%	3.18 (1.47)
Perpetration	121	3	2%	1	0	0%	NA
Stressful experience	121	72	60%	62	29	47%	4.21 (0.96)
Male non-veteran sample							
Any ALE	183	153	84%	137	44	32%^d^	4.14 (0.76)
Any severe ALE	183	96	52%	85	16	19%	4.35 (0.76)
Disaster	183	34	19%	30	0	0%	NA
Accident	183	51	28%	47	4	9%	3.00 (0.82)
Physically assaulted	183	51	28%	50	7	14%	4.43 (0.79)
Sexually assaulted	183	13	7%^b^	10	1	10%^d^	4.00 (NA)
Sex. uncomfortable	183	36	20%^b^	35	5	14%	3.80 (1.79)
Illness/Injury	183	25	14%	22	4	18%	5.00 (0.00)
Loss	183	62	34%	53	13	25%	4.08 (1.16)
Perpetration	183	13	7%	11	2	18%	4.50 (0.71)
Stressful experience	183	93	51%	87	29	33%	4.28 (0.79)

^a^ and ^b^ indicate statistically significant differences between women and men in ALE experience. ^c^ and ^d^ indicate significant differences between women and men in ALE reexperience. The sum of males and females does not always match the values in Table 1 due to one participant not disclosing their sex.

### Comparing the prevalence of adverse life events and reexperiencing between participants with versus without a self-reported lifetime diagnosis of PTSD

Participants with a lifetime PTSD diagnosis reported all ALEs with the exception of sexual assault and sexually uncomfortable experiences at significantly higher rates than participants without a PTSD diagnosis.

Participants with a PTSD diagnosis also exhibited a higher rate of reexperiencing ALEs (X2 = 6.83, p = 0.024), severe ALEs (X^2^ = 14.60, *p* < 0.001), disaster (X^2^ = 15.44, *p* < 0.001), accident (X^2^ = 8.80, *p* < 0.011), physical assault (X^2^ = 10.47, *p* = 0.004), illness/injury (X^2^ = 5.48, *p* = 0.045), loss (X^2^ = 8.33, *p* = 0.014), and perpetration (X^2^ = 13.35, *p* < 0.001).

**Figure 3:**
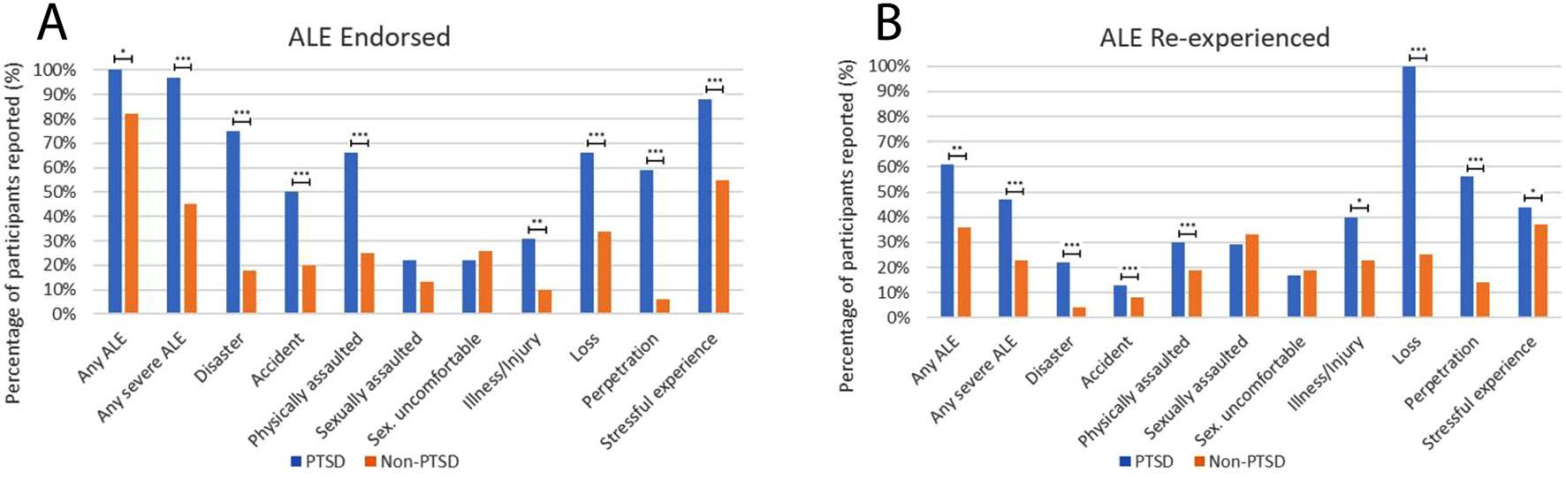
Percentage prevalence of ALE and ALE reexperiencing in participants with a lifetime PTSD diagnosis (n = 32) and without a lifetime PTSD diagnosis (n = 128). Plot (**A**) shows differences between subgroups in the prevalence of ALE experience. Plot (**B **) shows differences in prevalence of ALE re-experience. Asterisks indicate statistically significant differences: *p < 0.05, **p < 0.01, ***p < 0.005.

Figure 3, Table 3, and Table S2 present these results.

**Table 3:** Prevalence of adverse life event experience and adverse life event reexperience by lifetime PTSD diagnosis.

	Adverse life event experienced			Adverse life event reexperienced			
	N	Participants reporting	Percentage	N	Participants reporting	Percentage	Intensity mean (SD)
PTSD sample							
Any ALE	32	32	100%^a^	31	19	61%^c^	3.90 (1.08)
Any severe ALE	32	31	97%^a^	30	14	47%^c^	4.04 (1.21)
Disaster	32	24	75%^a^	23	5	22%^c^	3.00 (1.59)
Accident	32	16	50%^a^	15	2	13%^c^	5.00 (0.00)
Physically assaulted	32	21	66%^a^	20	6	30%^c^	3.80 (1.79)
Sexually assaulted	32	7	22%	7	2	29%	4.00 (2.00)
Sex. uncomfortable	32	7	22%	6	1	17%	NA
Illness/Injury	32	10	31%^a^	10	4	40%^c^	4.25 (1.50)
Loss	32	21	66%^a^	10	10	100%^c^	3.67 (1.41)
Perpetration	32	19	59%^a^	18	10	56%^c^	4.22 (1.56)
Stressful experience	32	28	88%^a^	27	12	44%	3.83 (1.19)
Non-PTSD sample							
Any ALE	128	105	82%^b^	100	36	36%^d^	3.89 (1.02)
Any severe ALE	128	58	45%^b^	57	13	23%^d^	4.08 (0.93)
Disaster	128	23	18%^b^	23	1	4%^d^	3.00 (NA)
Accident	128	26	20%^b^	26	2	8%^d^	4.00 (1.41)
Physically assaulted	128	32	25%^b^	31	6	19%^d^	4.00 (1.26)
Sexually assaulted	128	16	13%	15	5	33%	3.80 (1.30)
Sex. uncomfortable	128	33	26%	31	6	19%	3.33 (1.51)
Illness/Injury	128	13	10%^b^	13	3	23%^d^	5.00 (0.00)
Loss	128	44	34%^b^	40	10	25%^d^	3.70 (1.34)
Perpetration	128	8	6%^b^	7	1	14%^d^	5.00 (NA)
Stressful experience	128	71	55%^b^	67	25	37%	4.17 (0.87)

^a^ and ^b^ indicate statistically significant differences between participants with and without a self-reported lifetime PTSD diagnosis in ALE experience. ^c^ and ^d^ indicate significant differences between samples in ALE reexperience.

### Examining the association between acute factors and ALE reexperiencing

In the non-veteran sample, reexperiencing at least one ALE was significantly associated with *MEQ Mystical* (*r* = 0.18, *p* = 0.022), *AEI Reappraisal* (*r* = 0.24, *p* = 0.004), and *AEI Discomfort* (*r* = 0.33, *p* < 0.001), constructs reflecting mystical-type experience, introspective reappraising of difficult life experiences, and arduous acute experience, respectively. The number of distinct ALE types non-veterans reexperienced during ceremonies (*Reexperiencing Index*) was additionally associated with *AEI Reappraisal* (*r* = 0.18, *p* = 0.045) and *AEI Discomfort* (*r* = 0.25, *p* = 0.004).

In the veteran sample, reexperiencing at least one ALE was significantly associated with *AEI Clarity* (*r* = 0.52, *p* = 0.021) and *AEI Reappraisal* (*r* = 0.72, *p* < 0.001). Reexperiencing at least one severe ALE was significantly associated with *AEI Reappraisal* (*r* = 0.50, *p* = 0.028) *AEI Discomfort* (*r* = 0.55, *p* = 0.014). The number of distinct ALE types veterans reexperienced during ceremonies was associated with *AEI Reappraisal* (*r* = 0.57, *p* = 0.011) and *AEI Discomfort* (*r* = 0.58, *p* = 0.011).

In the non-veteran female sample, reexperiencing at least one ALE was significantly associated with *MEQ Mystical* (*r* = 0.29, *p* = 0.022), *AEI Clarity* (*r* = 0.30, *p* = 0.024), *AEI Reappraisal* (*r* = 0.36, *p* = 0.004), and *AEI Discomfort* (*r* = 0.30, *p* = 0.024). The number of distinct ALE types reexperienced during ceremonies was associated with AEI *Discomfort* (*r* = 0.27, *p* = 0.044).

In the non-veteran male sample, reexperiencing at least one ALE was significantly associated with only *AEI Discomfort* (*r* = 0.30, *p* = 0.004).

In the PTSD sample, reexperiencing at least one ALE (*r* = 0.44, *p* = 0.040), reexperiencing at least one severe ALE (*r* = 0.51, *p* = 0.019), and the number of distinct ALE types (*r* = 0.50, *p* = 0.017) were significantly associated with AEI Discomfort.

In the non-PTSD sample, reexperiencing at least one ALE was associated with AEI Reappraisal (*r* = 0.32, *p* = 0.008) and AEI Discomfort (*r* = 0.36, *p* < 0.001). Similarly, the number of distinct ALE types was associated with AEI Reappraisal (*r* = 0.28, *p* = 0.021) and AEI Discomfort (*r* = 0.28, *p* = 0.022).

Table 4 presents these results. Table S3 provides item-level correlations between *AEI Reappraisal* and *Discomfort* items and reexperiencing in the full sample.

**Table 4:** Association between acute factors and adverse life event reexperience.

	MEQ Mystical	AEI Clarity	AEI Reappraisal	AEI Discomfort
Non-veteran sample				
Any ALE	0.18*	0.10	0.24***	0.33***
Any severe ALE	0.12	0.04	0.08	0.10
Reexperiencing index	0.14	0.07	0.18*	0.25***
Veteran sample				
Any ALE	0.33	0.52*	0.72***	0.46*
Any severe ALE	0.34	0.38	0.50*	0.55*
Reexperiencing index	0.33	0.43	0.57*	0.58*
Female non-veteran sample				
Any ALE	0.29*	0.30*	0.36***	0.30*
Any severe ALE	0.16	0.20	0.12	0.13
Reexperiencing index	0.22	0.20	0.25	0.27*
Male non-veteran sample				
Any ALE	0.12	− 0.07	0.11	0.30***
Any severe ALE	0.07	− 0.13	− 0.06	0.04
Reexperiencing index	0.08	− 0.05	0.07	0.18
PTSD sample				
Any ALE	0.18	0.26	0.38	0.44*
Any severe ALE	0.04	0.07	0.15	0.51*
Reexperiencing index	0.18	0.25	0.32	0.50*
Non-PTSD sample				
Any ALE	0.17	0.15	0.32**	0.36***
Any severe ALE	0.08	0.17	0.30	0.06
Reexperiencing index	0.11	0.13	0.28*	0.28*

Values indicate correlations between acute factor variables and binary adverse life event reexperiencing variables. The Reexperiencing Index variable indexes the number of discrete ALE event types that participants reported. The size of the non-veteran sample ranged from 99 to 213; the size of the veteran sample ranged from 25 to 28; the size of the female sample ranged from 41 to 84; the size of the male sample ranged from 57 to 128; the size for PTSD sample ranged from 28 to 29; the size of the non-PTSD sample ranged from 49 to 92; *p < 0.05, **p < 0.01, ***p < 0.005.

### Examining whether change in Neuroticism is moderated by previous ALEs or ALE reexperiencing

Consistent with previous analyses^49^, the combined sample exhibited a significant reduction in *Neuroticism*. Participants showed a decline of 0.53 units (on 5-point Likert-scale) in *Neuroticism* between baseline and post, and a decline of 0.47 units between baseline and follow-up.

Eleven models were subsequently conducted testing the moderating influence of ALE experience and reexperience variables on estimated changes in Neuroticism between baseline and subsequent timepoints. Results from these models can be found in Table 5 and detailed results can be found in Table S4.

**Table 5:** Examining moderation of changes in Neuroticism by adverse life event experience and reexperience in the combined sample.

Moderator	Between baseline & post		Between baseline and 3-month follow-up		Sample size B
	Timepoint B	Interaction Term B	Timepoint B	Interaction Term B	
(A) Adverse life event experienced (full sample)					
Any ALE	− 0.51	− 0.02	− 0.41	− 0.07	312
Any severe ALE	− 0.54	0.02	− 0.47	0.01	312
Adverse life event index	− 0.49	− 0.02	− 0.41	− 0.02	312
(B) Adverse life event reexperienced (full sample)					
Any ALE	− 0.45	− 0.21***	− 0.39	− 0.19**	301
Any severe ALE	− 0.50	− 0.18*	− 0.44	− 0.14	301
Reexperiencing index	− 0.47	− 0.06***	− 0.42	− 0.05*	301
(C) Adverse life event reexperienced (only participants who endorsed previous ALE)					
Any ALE (combined sample)	− 0.46	− 0.18*	− 0.40	− 0.19*	259
Any ALE (female sample)	− 0.38	− 0.24*	− 0.37	− 0.19	93
Any ALE (male sample)	− 0.49	− 0.16*	− 0.41	− 0.19*	166
Any severe ALE (combined sample)	− 0.47	− 0.17	− 0.41	− 0.16	170
Reexperiencing index (combined sample)	− 0.48	− 0.05*	− 0.42	− 0.05*	259

B = unstandardized coefficient. These results reflect eleven linear mixed effects models in which ALE experience and ALE reexperience variables were tested as moderators of the effect of Timepoint on Neuroticism (i.e., change in Neuroticism). Coefficients are unstandardized, and the Interaction Term coefficients represent the effect of interactions between each moderator and Timepoint on Neuroticism scores, (i.e., the estimated effect of a one-unit increase in the moderator on estimated change in Neuroticism between two timepoints). The moderators were unstandardized in these models.

Section A reflects moderation of change in Neuroticism by ALE experience variables in the full sample. Section B reflects moderation of Neuroticism by ALE reexperience variables in the full sample. Section C reflects moderation of Neuroticism by ALE reexperience variables among only participants who endorsed previous ALE history. Results from two additional analyses are also included that focus on females and males separately. The effect of Timepoint was not inferentially interpreted.

*p < 0.05, **p < 0.01, ***p < 0.005.

As presented in Table 5 Section A, none of the three tested ALE experience variables did not significantly moderate change between baseline and subsequent timepoints.

As presented in Table 5 Section B, using data from the full sample (i.e., not only participants who endorsed previous ALEs), reexperiencing one or more ALEs showed evidence of potentiating reductions in *Neuroticism*, such that reexperiencing was associated with an incremental 0.21 and 0.19 unit decrease in *Neuroticism* between baseline and post (*p* = 0.003), and baseline and follow-up (*p* = 0.009), respectively. Reexperiencing one or more severe ALEs similarly showed evidence of potentiating reductions in *Neuroticism* between baseline and post (B = 0.012, *p* = 0.031). Lastly, reexperiencing an additional ALE event type (represented by the *Reexperiencing Index* moderator) was associated with an incremental 0.06 unit decrease in *Neuroticism* between baseline and post (*p* = 0.004), and 0.05 between baseline and follow-up (*p* = 0.022).

As a caveat, participants were included in this set of analyses who did not endorse previous ALEs, but nevertheless reported reexperiencing previous ALEs. These individuals were believed to have either temporarily forgotten their previous ALE history at baseline (or neglected to report them), experienced false memories during ceremony, or reexperienced repressed memories during ceremony.

As presented in Table 5 Section C, analyses were also conducted using data from only participants who previously endorsed ALEs. Here, the combined sample was examined along with female and male subsamples. Results reflected a similar pattern to the above.

**Figure 4:**
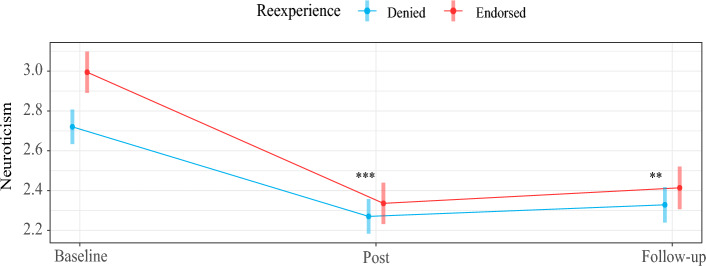
The plot shows the degree to which, in the full sample, reexperiencing during ceremony was associated with a greater decline in Neuroticism. Asterisks indicate significant moderation of change in Neuroticism by reexperiencing: **p < 0.01, ***p < 0.005.

Figure 4 illustrates the pattern of moderation of change in *Neuroticism* by reexperiencing in the full sample; other instances of significant moderation noted above reflected the same pattern.

### False discovery rate results

The False Discovery Rate adjustment was applied to one-hundred-sixty p-values (corresponding to main analyses). A false discovery rate of 1.78% was observed, and adjusted p-values reflected an estimated 71 rejections of the null hypothesis at *p* < 0.05. Results within the main text reflect these adjusted p-values.

## Discussion

Using a large sample of ayahuasca retreat-goers, the present study set out to investigate whether ceremonial ayahuasca promotes autobiographical reexperiencing of adverse life events, how well reexperiencing can be predicted from individual characteristics, and the impact of reexperiencing on mental health. Understanding psychedelic-induced autobiographical reexperiencing holds substantial clinical relevance as recollections of adverse or traumatic events could pose psychological harm under unsupportive conditions, and/or may hold therapeutic value within the context of trauma-focused psychedelic treatment.

### Does psychedelic experience in the ceremonial ayahuasca context amplify autobiographical reexperiencing of adverse life events?

Our principle finding was that reexperiencing adverse life events was common among all participants, with four in ten non-veteran participants reporting reexperiencing of adverse life events, and over a quarter reporting reexperiencing of more severe adverse life events, i.e., excluding stressful experiences and sexually uncomfortable experiences. These observations are consistent with observations from clinical trials^17,18,20^ and naturalistic studies^21,22^ of autobiographical reexperiencing under psilocybin and ayahuasca. Participants notably rated their reexperiences at a relatively high intensity (i.e., average rating of 3.94 out of 5 for adverse life events), which is consistent with Carhart-Harris and colleagues’^12^ observations of functional hyperactivation of visual and sensory brain areas and vivid subjective recollections under LSD when cued to recollect autobiographical memories.

Our observations are the first to show the relative prevalence of different types of adverse life event reexperiencing. Approximately a quarter or more of non-veteran participants reexperienced sexual assault, sexually uncomfortable events, loss of significant others, and general stressful experiences.

The contents of participants’ reexperiences was not precisely examined in our study, but participants who reported reexperiencing were more likely to report experiences of discomfort, as well as constructive engagement with challenging emotional material during ceremony (i.e., reappraisal, psychological flexibility), including discovering positive meaning in past trauma and feeling gratitude for previous life challenges. Although these associations could be suggestive that psychedelic reexperiencing is generally characterized by qualities of reappraisal and discomfort, it could also be the case that psychedelic experiences of reappraisal and discomfort prime the emergence of adverse life event recollections. The latter possibility is supported by evidence that negative mood states promote mood-congruent (i.e., negative) memories (^63,64^;c.f. ^65^), and the fact that acute experiences of reappraisal and discomfort were measured globally (i.e., degree of acute experience), whereas reexperiencing was measured discretely (i.e., presence or absence of discrete episodes).

Given that reexperiencing has been associated in the literature with multiple substances that share serotonergic 2A agonism as a cellular mechanism of action, we believe the present findings respecting ayahuasca are likely to generalize to other classic psychedelic substances. Nevertheless, other factors associated with the use of ayahuasca may influence the emergence of autobiographical memories, including ceremonial elements, group context, molecules unique to ayahuasca (e.g., harmine), and/or spiritual forces (recognized within Shipibo metaphysics).

### Do certain populations reexperience at a higher probability?

Our results pointed to two major observations regarding which populations may possess a higher probability of reexperiencing. First, women exhibited a greater likelihood of reexperiencing adverse life events than men, despite an equal rate of overall previous adverse life events between groups. This result was substantively driven by differences between females and males in reexperiencing sexual assault, with four in ten women with previous history of sexual assault recollecting their assault experiences compared to just one in ten men doing so. It is not clear what factors account for an overall higher reexperiencing rate among women, but our analyses indicated that reexperiencing women tended to report more mystical-type experience and cognitive reappraisal during ceremony than non-reexperiencing women with a similar adverse life event history, whereas men did not exhibit this pattern. These observations are the first to show differential risk of reexperiencing among women versus men, and should inform future protocols for psychedelic therapy, as will be explored below.

Second, veterans exhibited a greater likelihood of reexperiencing certain adverse life events than non-veterans. These events included memories of disasters, loss of significant others, and episodes of harming others. Veterans with special operations forces and combat experiences possess a trauma profile involving loss of significant others, personal injuries, as well as episodes of inflicting harm on others in addition to being harmed themselves^10,27^. Our data were suggestive that these types of life events were substantially more likely to emerge during ceremony, with more than 50% of veterans with a trauma history recollecting episodes of adverse life events in general, and illness or injury, loss, and harming others, specifically. This pattern is suggestive both of risks presented by psychedelic treatments to the veteran community (and perhaps especially those with a history of combat-exposure and special operations service), as well as opportunities for constructive emotional processing under appropriate therapeutic conditions. Relevant to risk, in the present sample, reexperiencing veterans were more likely than reexperiencing non-veterans to experience discomfort within their ceremonies. Relevant to treatment, reexperiencing veterans were more likely to engage in cognitive reappraisal and psychological flexibility. Finally, our supplementary analyses were suggestive that for veterans, being higher in neuroticism was associated with a greater probability of reexperiencing. Clinicians may view this as a risk-factor or a promotive-factor depending on treatment focus.

Third, participants with a self-reported lifetime diagnosis of PTSD exhibited a greater likelihood of reexperiencing most adverse life events than participants without a lifetime diagnosis. These events included memories of disasters, accidents, physical assaults, illnesses or injuries, loss of significant others, and episodes of harming others. Our data were suggestive that these types of life events were substantially more likely to emerge during ceremony, with more than 60% of participants with a lifetime diagnosis recollecting episodes of adverse life events in general. These results are perhaps not surprising given that a diagnosis of PTSD involves persisting attention to previous trauma, impacts on self-, other-, and world-beliefs, and sensitivity to trauma-related cues.

### Does reexperiencing confer therapeutic benefit?

A substantive body of qualitative research presents accounts by psychedelic users showing that psychedelic reexperiencing of past trauma may be promotive of healing, and our qualitative findings were consistent with this. Individuals who recollected adverse life events during ceremony were more likely to report decreased neuroticism both immediately following ceremonies as well as three months later.

Conservatively, we conclude that psychedelic-induced change in neuroticism was invariant to reexperiencing adverse life events, with one possible implication being that psychedelic reexperiencing of traumatic events does not appear to interfere with the therapeutic effect of ayahuasca. Previous history of adverse life events was similarly not related to outcome, suggesting that the therapeutic effect of ayahuasca ceremony and psychedelics more broadly is not augmented or reduced by an individual’s previous trauma history.

A more liberal interpretation would be that our data is the first to systematically demonstrate the association between psychedelic reexperiencing and positive mental health outcomes. It is conceivable that the cognitive mechanisms underlying these changes involve ways of relating to trauma and adverse life events under ayahuasca that are distinctly therapeutic. For example, our results demonstrated that our *reappraisal* factor was generally associated with reexperiencing, raising the possibility that reappraisal represents one such mechanism. Item level analyses foregrounded the following items (in order of correlational magnitude, Table S3) as showing moderate associations with reexperiencing:

1. I was able to see new positive meaning in a past trauma (*r* = 0.39)
2. I felt that I was forced to confront negative perceptions I've had of myself (*r* = 0.37)
3. I wrestled with my inner conflicts (*r* = 0.34)
4. I identified aspects of myself that cause me pain (*r* = 0.30)
5. I faced my fears (*r* = 0.29)
6. I considered that I am too hard on myself (*r* = 0.26)
7. Laughter or humor helped me overcome conflicts, fears, or difficult past experiences (*r* = 0.26)

Some of these elements are common to trauma-focused therapies recommended by the American Psychological Association, such as *Cognitive-Processing Therapy*^39^ and *Prolonged Exposure Therapy*, in which patients work toward displacing self-blame (e.g., addressing assimilation), and contemplating aversive memories and self-schemas without a sympathetic stress response. The first and last item, however, may provide a glimpse into what may be unique to psychedelic reexperiencing (though more research is needed, and meaning-making can emerge within the context of conventional trauma-focused therapies). These items along with item 6 are indicative of holding different perspectives on previous traumas such that the original negative meaning is not quite as resonant.

It is worth considering the cognitive states that would support this way of relating to previous hardship. Two possibilities include psychedelic-induced^1^ disposition toward connectedness versus separateness and ^2^ enhanced metacognition. Connectedness describes states of consciousness including connection to self, others, and world^66^, unitive consciousness involving merging with a larger whole^67^, alterations to attachment^68^, nature-relatedness^69^, and absorption in external phenomena^70^. Although connectedness is generally discussed in terms of these discrete phenomena, it is conceivable that these represent states of consciousness afforded by an underlying cognitive substrate involving a heightened capacity to recognize the meaningfulness of (or ascribe meaning to) inner and external phenomena. It is accordingly plausible that within psychedelic reexperiencing, adverse life events are themselves connected to in a new way, for example, in the form of viewing positive meaning in them and in oneself.

Metacognition describes the capacity of individuals to process and attend to their own mental states, including patterns of thoughts, emotions, as well as mental states underlying behavior^71,72^. Researchers have found evidence that ayahuasca and other psychedelics promote decentering^73–75^, involving the ability to observe thoughts and feelings as mental events (rather than representations of reality), and disidentify from internal experience, affording non-reactivity. Decentering in the context of reexperiencing may enable individuals to invalidate incorrect or unhelpful schemas about self, others, and world, and to hold new perspectives. For example, an individual reflecting on an episode of sexual assault may grapple with the adequacy of long-held beliefs about their blameworthiness for placing themselves in the situation, or may question the reasonableness of their generalized perception of threat in the external environment. It is likely that metacognitive processes of decentering support psychological flexibility, which has shown evidence of psychedelic-induced enhancement, both acutely^34^ and post-acutely^35,36,76^. Psychological flexibility in the context of reexperiencing may enable individuals to flexibly consider challenging beliefs about self and world in the service of reaching more adaptive perspectives, e.g., containing self-forgiveness and -compassion, resolving anger/fear, and honoring the formidable challenges they have faced.

An additional consideration pertinent to the therapeutic value of reexperiencing is whether the processes underlying the emergence of vivid psychedelic reexperiencing of genuine past events also underlie mistaken recollections of events that never took place but nevertheless emerge vividly in consciousness. As other scholars have suggested^77^, this latter category of reexperiencing warrants sensitive treatment from therapists, as dismissing possibly false memories that pose psychological challenge could provoke emotionally destabilizing consequences. Although our data cannot speak directly to the emergence of false or repressed memories, it bears noting that approximately 3% of participants in our sample claimed to reexperience adverse life event types that they did not report having experienced prior to their ceremonies (see Table S5).

### Implications for therapeutic practice

The present results hold implications for the preparation, administration, and integration phases of psychedelic therapy. In the preparation phase, we believe it will be important for researchers and clinicians to prepare subjects/patients for the possibility of reexperiencing adverse life events in the course of informed consent. Numerous scholars have advocated for a comprehensive and supportive consent process including detailed psychoeducation regarding the range of experiences that can result from psychedelic compounds^78–81^. The present results support Smith and Appelbaum’s^3^ call for explicit reference to the potential for re-exposure to trauma within the informed consent process. Furthermore, given that women, veterans, and individuals with a lifetime PTSD diagnosis were disproportionately more likely to encounter certain adverse life events, it may be important to share the heightened risk of re-exposure with these populations, particularly. From a research perspective, it may also be conditionally prudent to screen out certain participants with particular types of trauma history from participation (e.g., sexual assault trauma).

In the administration and integration phases, we believe there is value in researchers and clinicians being prepared with trauma-focused therapeutic skills regardless of the psychiatric condition under examination. These skills may enable clinicians to productively engage with subjects/patients’ trauma memory structures and stuckpoints. Indeed, although episodes of psychedelic reexperiencing show increasing evidence of being therapeutically constructive, more distressed accounts of reexperiencing have also been reported^77^, raising the potential value of applying trauma-focused skills. In addition, skills in trauma-focused modalities such as *Prolonged Exposure Therapy* and *Cognitive Processing Therapy* could be additionally specialized to the population being seen, as our observations point to a higher probability of reexperiencing sexual assault among women, and of reexperiencing disaster, accident, loss, physical assault, illness/injury, and episodes of harming others among combat-veterans.

### Limitations

A number of limitations should be noted. First, any claims regarding increased reexperiencing under ayahuasca should be qualified by the lack of a control condition, without which it cannot be known the rate of reexperiencing over the course of many hours of contemplative time. Mitigating this validity threat were participants’ high intensity ratings, which we regard as being suggestive of a pharmacological origin. Second, it should be stated that trauma, i.e., related to death, threatened death, actual or threatened serious injury, or actual or threatened sexual violence by DSM-5, was not formally assessed in this study, and our results, related more broadly to adverse life events, are not expected to generalize perfectly to populations with formal trauma history. Third, the validity of the present findings rest on the absence of memory biases related to psychedelic cognitive states. Future research on the reliability of reporting on acute phenomena is encouraged. However, evidence of post-acute memory deficits have not been shown, and high average intensity ratings of reexperiences may at least be suggestive of vividness and reliability. Fourth, one caution regarding the observed sex difference in reexperiencing is that men presented with lower rates of sexual assault history relative to women (N = 10 vs N = 32), making the reexperiencing estimate for men less reliable, and the significant sex difference itself deserving of replication.

## Supplementary Information

Supplementary Information.

## Data Availability

Data has been made available at https://osf.io/uxqcv/.
