# Supplementary material for: Prevalence and therapeutic impact of adverse life event reexperiencing under ceremonial ayahuasca
Source: Sci Rep. 2023 Jun 9;13:9438. doi: 10.1038/s41598-023-36184-3 (PMC10256717; doi:10.1038/s41598-023-36184-3)
Supplement: Supplementary file 1 — Supplementary Information. [file 41598_2023_36184_MOESM1_ESM.docx]

**Prevalence and therapeutic impact of adverse life event reexperiencing under ceremonial ayahuasca**

**Supplementary Materials**

Brandon Weiss PhD^a^ (bw64357@gmail.com)

Aleksandra Wingert MSc^a^ (a.wingert22@imperial.ac.uk)

David Erritzoe MD PhD^a^ (d.erritzoe@imperial.ac.uk)

W. Keith Campbell PhD^b^ (wkc@uga.edu)

a Imperial College London, Division of Psychiatry, London, UK

b University of Georgia, Athens, GA, USA

**Supplementary Materials I. Supplementary Tables referenced in the Main Text**

Tables S1 to S5 are referenced within the main text and provide supplementary information regarding the prevalence of adverse life event experience and adverse life event reexperience by sample and sex (Table S1), chi-square differences in ALE experience and reexperience between subgroups and between males and females (Table S2), item-level correlations between AEI Reappraisal, AEI Discomfort, and Reexperiencing any ALE (Table S3), tests of whether change in Neuroticism is moderated by ALE experience and reexperience (Table S4), and the number of participants who endorsed reexperiencing despite not previously endorsing ALE experience at baseline (Table S5).

**Supplementary Materials II. Psychometric properties of the Ayahuasca Experience Inventory (AEI)**

The AEI is not yet published, as more data is collected with the purpose of conducting a formal validation analysis. However, exploratory factor analyses have been conducted (1, 2), yielding a three-factor structure used in the present study, and evidence of convergent validity (3) and criterion validity (2) has been shown in previous studies using a similar sample. With respect to convergent validity, AEI Clarity has demonstrated considerable overlap with mystical experience (3). AEI Reappraisal has not yet shown convergence with other like measures, in part due to its distinctiveness, but we have future interest in examining associations with acute measures of insight. AEI Discomfort is considered face-valid, but demonstrating its convergence with measures indexing acute challenging experiences remains needed. With respect to criterion validity, AEI Clarity and AEI Reappraisal showed strongest evidence of being associated with adaptive personality changes in previous work. More work is needed to refine the factor structure, item composition, and construct validity of the AEI, but there is already considerable support for its adequacy as a useful acute measure. This is especially the case in the present context where it is being principally used to understand the nature of cognitive experience accompanying reexperiencing during ayahuasca ceremony.

**Supplementary Materials III. Examining whether change in Neuroticism differs for veterans versus non-veterans**

Little evidence of moderation of change in *Neuroticism* by sample emerged. Between baseline and post, veterans exhibited a nominally greater decline in *Neuroticism* of .06 units compared to non-veterans (*p* = .497), and between baseline and follow-up, veterans exhibited a nominally greater decline of .05 units compared to non-veterans (*p* = .590) (Table S6).

**Supplementary Materials IV. Predicting ALE reexperiencing from demographics, baseline personality traits, and pre-ceremony intentions**

Correlations were calculated between demographics, baseline personality domains, and pre-ceremony intentions, on one hand, and reexperiencing variables, on the other. See Table S7.

With respect to pre-ceremony intention data (i.e., *Trauma Healing Intention*, *Healing Intention*), of the 303 participants who provided reexperiencing data, 283 participants provided open-ended responses on their pre-ceremony intentions. Of these, 143 (51%) reported a healing intention, and 26 (9%) reported an intention focused on healing trauma specifically.

As a reminder, at post-retreat assessment, participants were asked to describe their pre-ceremony intentions open-endedly (“What intentions did you bring into your ceremonies?”). Responses were then coded for whether they contained intentions related to either resolving stress surrounding previous ALEs (*Trauma Healing Intention*) or more general mental health-related healing (*Healing Intention*). The first and second authors both performed this coding, and deliberated in cases of disagreement to reach consensus. The *Trauma Healing Intention* and *Healing Intention* variables were categorical and binary, with each participant scoring either 0 (absent) or 1 (present).

**Supplementary Materials V. Examining qualitative reflections on reexperiencing**

Nineteen participants reflected on the influence of their ceremony experiences on their relationship to previous traumas. There were three notable characteristics of these reflections. First, participants reported relief from tension and stress attributed to traumatic life experiences:

[The] [m]ajority of my emotional toxicity from childhood trauma is gone, I feel incredible.

[I]t allowed me to learn lessons that I before saw as traumatic and sad but now [see] as beautiful lessons.

I have a general ease, comfort, and direction. I value myself more, [I’ve] lost tension and trauma held physically in my muscles.

I don't overthink anymore after the ayahuasca experience and I have the feeling that a lot of the trauma I had, left my body during the ayahuasca experience.

[I was successful in] letting go of a past trauma, that it no longer matters in the same way and I don't have to keep revisiting it. A sense of freedom from that trauma. Freedom of movement and energy flow. That my body isn't my enemy anymore.

A second theme involved actively processing and thoughtfully reconsidering their relationship to previous trauma. These participants did not claim full relief, but shared their intention to initiate actions, emotional or physical, in the service of continued healing:

The core trauma that was brought up in my ceremonies was related to my parents (who also received this survey). This has been more difficult to communicate with them and work on. I feel like it will happen, but will take time.

I got to let go of my past and gain back a part of myself that had gone missing due to a childhood trauma. That trauma had a large impact on my life, specifically my relationship with my parents and my ex-wife. Understanding, processing, and learning from that experience really opens me up and I'm excited about my future.

My intention in my ceremonies was to heal from trauma. Since then I have been learning more about my past and am tackling these things. Emotionally, it is hard work but it is important and I am choosing to face it. My answers reflect a period when I am facing these things head on.

Finally, one participant observed increases in their empathy and understanding for the ways in which individuals’ trauma influences their actions:

My understanding that other people have their own trauma which affects the way they behave, was increased by the ayahuasca and now I feel less judgmental and more understanding and patient.

**Supplementary Tables**

**Table S1**

Prevalence of adverse life event experience and adverse life event reexperience by sample and sex

|  | Adverse Life Event Experienced | | | | | | Adverse Life Event Reexperienced | | | | | |
| --- | --- | --- | --- | --- | --- | --- | --- | --- | --- | --- | --- | --- |
|  | Males N | Males reporting | Percentage | Females N | Females reporting | Percentage | Males N | Males reporting | Percentage | Females N | Females reporting | Percentage |
| Non-veteran sample |  |  |  |  |  |  |  |  |  |  |  |  |
| Any ALE | 183 | 153 | 84% | 121 | 106 | 88% | 137 | 44 | 32% | 90 | 51 | 57% |
| Any severe ALE | 183 | 96 | 52% | 121 | 65 | 54% | 85 | 16 | 19% | 53 | 22 | 42% |
| Disaster | 183 | 34 | 19% | 121 | 18 | 15% | 30 | 0 | 0% | 13 | 1 | 8% |
| Accident | 183 | 51 | 28% | 121 | 25 | 21% | 47 | 4 | 9% | 20 | 0 | 0% |
| Physically assaulted | 183 | 51 | 28% | 121 | 27 | 22% | 50 | 7 | 14% | 21 | 6 | 29% |
| Sexually assaulted | 183 | 13 | 7% | 121 | 39 | 32% | 10 | 1 | 10% | 32 | 13 | 41% |
| Sex. uncomfortable | 183 | 36 | 20% | 121 | 63 | 52% | 35 | 5 | 14% | 52 | 16 | 31% |
| Illness/Injury | 183 | 25 | 14% | 121 | 13 | 11% | 22 | 4 | 18% | 12 | 2 | 17% |
| Loss | 183 | 62 | 34% | 121 | 45 | 37% | 53 | 13 | 25% | 39 | 10 | 26% |
| Perpetration | 183 | 13 | 7% | 121 | 3 | 2% | 11 | 2 | 18% | 1 | 0 | 0% |
| Stressful experience | 183 | 93 | 51% | 121 | 72 | 60% | 87 | 29 | 33% | 62 | 29 | 47% |
| Veteran sample |  |  |  |  |  |  |  |  |  |  |  |  |
| Any ALE | 30 | 30 | 100% | 3 | 3 | 100% | 29 | 18 | 62% | 3 | 1 | 33% |
| Any severe ALE | 30 | 30 | 100% | 3 | 3 | 100% | 29 | 14 | 48% | 3 | 1 | 33% |
| Disaster | 30 | 26 | 87% | 3 | 1 | 33% | 25 | 6 | 24% | 1 | 0 | 0% |
| Accident | 30 | 18 | 60% | 3 | 0 | 0% | 17 | 3 | 18% | 0 | 0 | 0% |
| Physically assaulted | 30 | 18 | 60% | 3 | 2 | 67% | 17 | 5 | 29% | 2 | 1 | 50% |
| Sexually assaulted | 30 | 2 | 7% | 3 | 3 | 100% | 2 | 0 | 0% | 3 | 0 | 0% |
| Sex. uncomfortable | 30 | 3 | 10% | 3 | 3 | 100% | 3 | 0 | 0% | 3 | 1 | 33% |
| Illness/Injury | 30 | 25 | 83% | 3 | 0 | 0% | 12 | 6 | 50% | 0 | 0 | 0% |
| Loss | 30 | 23 | 77% | 3 | 2 | 67% | 22 | 12 | 55% | 2 | 1 | 50% |
| Perpetration | 30 | 19 | 63% | 3 | 1 | 33% | 18 | 9 | 50% | 1 | 1 | 100% |
| Stressful experience | 30 | 27 | 90% | 3 | 2 | 67% | 26 | 13 | 50% | 2 | 0 | 0% |
| *Note.* ALE = adverse life event. For significant differences in prevalence between non-veterans and veterans, and between males and females, see Table S2. | | | | | | | | | | | | |

**Table S2**

Chi-square tests of differences in ALE experience and reexperience between subgroups

|  |  | Adverse life event experienced | | | | | | Adverse life event reexperienced | | | | | |
| --- | --- | --- | --- | --- | --- | --- | --- | --- | --- | --- | --- | --- | --- |
|  |  | Non-veterans vs veterans | | Males vs Females | | PTSD vs Non-PTSD | | Non-veterans vs veterans | | Males vs Females | | PTSD vs Non-PTSD | |
|  |  | X^2^ | p-value (BH adjusted) | X^2^ | p-value (BH adjusted) | X^2^ | p-value (BH adjusted) | X^2^ | p-value (BH adjusted) | X^2^ | p-value (BH adjusted) | X^2^ | p-value (BH adjusted) |
|  | Any ALE | 4.53 | .033 (.069) | .63 | .426 (.509) | 5.33 | .021 (.048) | 4.05 | .044 (.084) | 9.99 | .002 (.008) | 6.83 | .009 (.024) |
|  | Any severe ALE | 25.11 | .000 (.000) | .01 | .922 (.958) | 25.52 | .000 (.000) | 12.09 | .001 (.004) | 4.52 | .033 (.069) | 14.60 | .000 (.000) |
|  | Disaster | 66.46 | .000 (.000) | .47 | .494 (.569) | 37.43 | .000 (.000) | 37.34 | .000 (.000) | 1.12 | .289 (.376) | 15.44 | .000 (.000) |
|  | Accident | 11.68 | .001 (.004) | 1.65 | .199 (.274) | 10.17 | .001 (.004) | 18.80 | .000 (.000) | .16 | .688 (.754) | 8.80 | .003 (.011) |
|  | Physically assaulted | 15.80 | .000 (.001) | .91 | .341 (.430) | 17.28 | .000 (.000) | 13.73 | .000 (.001) | .07 | .786 (.842) | 10.47 | .001 (.004) |
|  | Sexual assault | .00 | .981 (.983) | 30.69 | .000 (.000) | 132.00 | .964 (.976) | 1.86 | .172 (.249) | 14.73 | .000 (.001) | 132.00 | .964 (.976) |
|  | Sexual discomfort | 2.17 | .140 (.211) | 33.35 | .000 (.000) | 132.00 | .964 (.976) | .82 | .366 (.447) | 5.07 | .024 (.053) | 132.00 | .964 (.976) |
|  | Illness/Injury | 11.75 | .001 (.004) | .33 | .565 (.641) | 7.62 | .006 (.019) | 20.32 | .000 (.000) | .03 | .870 (.916) | 5.48 | .019 (.045) |
|  | Loss | 18.79 | .000 (.000) | .22 | .639 (.710) | 9.11 | .003 (.011) | 24.25 | .000 (.000) | .57 | .449 (.532) | 8.33 | .004 (.014) |
|  | Perpetration | 90.49 | .000 (.000) | 2.27 | .132 (.201) | 47.79 | .000 (.000) | 36.23 | .000 (.000) | .48 | .490 (.568) | 13.35 | .000 (.000) |
|  | Stressful exp | 12.68 | .000 (.002) | 1.88 | .171 (.249) | 9.82 | .002 (.008) | 2.41 | .121 (.190) | 2.31 | .128 (.197) | 3.51 | .061 (.109) |
|  | *Note.* Differences between males and females were only examined in non-veteran sample due to a small sample size of women in veteran sample (n=3). BH = Benjamini and Hochberg’s (1995) False Discovery Rate adjusted p-values. | | | | | | | | | | | | |

**Table S3.**

Item-level correlations between AEI Reappraisal, AEI Discomfort, and Reexperiencing any ALE

| AEI Subscale | Item | Reexperience |
| --- | --- | --- |
| AEI Reappraisal | I was able to see new positive meaning in a past trauma | .39** |
|  | I felt that I was forced to confront negative perceptions I've had of myself | .37** |
|  | I wrestled with my inner conflicts | .34** |
|  | I identified aspects of myself that cause me pain. | .30** |
|  | I faced my fears | .29** |
|  | I considered that I am too hard on myself. | .26** |
|  | Laughter or humor helped me overcome conflicts, fears, or difficult past experiences. | .26** |
|  | I felt that I missed loved ones greatly. | .26** |
|  | I realized how critical it is that I make changes in my life. | .23** |
|  | I overcame/resolved my fears | .22** |
|  | I realized that I can just be, without obligation or pressure to act in a particular way | .22** |
|  | Felt gratitude for the little or previously insignificant moments in my life | .22** |
|  | Felt gratitude for the love and support of those around me | .22** |
|  | I felt forgiveness | .21** |
|  | I felt deep compassion for people who have wronged me or people close to me | .19* |
|  | I realized that I am unnecessarily bound to repeat patterns of acting/feeling/behaving | .19* |
|  | Engaged in problem solving about how to communicate with someone. | .19* |
|  | I saw the humor in situations that I previously may have taken too seriously | .18' |
|  | I experienced deep compassion for others | .18' |
|  | I realized how courageous I am. | .17' |
|  | I felt capable of living courageously | .17' |
|  | Felt gratitude for the lessons I learned from people that had wronged me | .17' |
|  | I felt great compassion for others' distress or pain. | .16' |
|  | I recognized that I can live more freely, without a sense of obligation. | .15' |
|  | I related to my physical pain with a sense of braveness | .13 |
|  | I felt courageous | .12 |
|  | Felt a disappearance of opposing forces such as good and bad, right and wrong. | .11 |
|  | I realized that I am unnecessarily bound to follow social norms | .09 |
|  | Felt gratitude for the challenges life had given me | .09 |
|  | I felt that I have great potential in me to achieve what I need in life. | .06 |
| AEI Discomfort | I felt agony. | .38** |
|  | I experienced my surroundings as strange and weird. | .28** |
|  | I experienced everything as frighteningly distorted. | .27** |
|  | I felt overwhelmed by the experience | .27** |
|  | I felt threatened. | .25** |
|  | I felt as if dark forces had overtaken me. | .25** |
|  | I felt lost | .25** |
|  | I felt exhausted. | .24** |
|  | I felt tormented. | .24** |
|  | I experienced great physical discomfort | .24** |
|  | I felt an unpleasant flow of information that I couldn't control | .24** |
|  | Time passed slowly in a tormenting way. | .23** |
|  | I stayed frozen in an very unnatural position for an extended period of time. | .22** |
|  | I felt isolated from everything and everyone. | .21** |
|  | I was afraid that the state I was in would last forever. | .17' |
| *Note.* AEI = Ayahuasca Experience Inventory; ALE = Adverse life event; N ranged from 195 to 198. Only participants who had previously endorsed ALEs were included in these analyses. ‘*p*<.05, **p*<.01, ***p*<.005 | | |

**Table S4.**

Linear mixed effects models regressing Neuroticism onto the interaction between Time and ALE experience and reexperience

moderators

| ALE Experiencing (N = 312) | | | | | |
| --- | --- | --- | --- | --- | --- |
| Moderator | Parameter | B | Std.Error | t-value | p-value (BH adjusted) |
| Any ALE | Intercept | 2.85 | .14 | 2.50 | .000 |
|  | Any ALE | .07 | .10 | .75 | .455 |
|  | Time Post | -.51 | .08 | -6.69 | .000 |
|  | Time Follow-up | -.41 | .08 | -5.26 | .000 |
|  | Age | .00 | .00 | -.81 | .419 |
|  | Any ALE x Time Post | -.02 | .08 | -.27 | .789 (.842) |
|  | Any ALE x Time Follow-up | -.07 | .08 | -.83 | .409 (.496) |
| Any adverse ALE | Intercept | 2.90 | .12 | 24.61 | .000 |
|  | Any adverse ALE | .01 | .07 | .20 | .841 |
|  | Time Post | -.54 | .04 | -12.38 | .000 |
|  | Time Follow-up | -.47 | .04 | -1.60 | .000 |
|  | Age | .00 | .00 | -.82 | .413 |
|  | Any adverse ALE x Time Post | .02 | .06 | .29 | .774 (.837) |
|  | Any adverse ALE x Time Follow-up | .01 | .06 | .12 | .901 (.942) |
| Index | Intercept | 2.90 | .12 | 24.56 | .000 |
|  | Index | .00 | .02 | .07 | .941 |
|  | Time Post | -.49 | .05 | -1.59 | .000 |
|  | Time Follow-up | -.41 | .05 | -8.67 | .000 |
|  | Age | .00 | .00 | -.75 | .456 |
|  | Index x Time Post | -.02 | .01 | -1.08 | .279 (.366) |
|  | Index x Time Follow-up | -.02 | .02 | -1.45 | .147 (.218) |
| Reexperiencing (Full Sample) (N = 301) | | | | | |
| Moderator | Parameter | B | Std.Error | t-value | p-value (BH adjusted) |
| Any ALE | Intercept | 2.80 | .12 | 23.87 | .000 |
|  | Any ALE | .27 | .07 | 3.87 | .000 |
|  | Time Post | -.45 | .04 | -11.96 | .000 |
|  | Time Follow-up | -.39 | .04 | -1.02 | .000 |
|  | Age | .00 | .00 | -.75 | .454 |
|  | Any ALE x Time Post | -.21*** | .06 | -3.49 | .001 (.003) |
|  | Any ALE x Time Follow-up | -.19** | .06 | -3.05 | .002 (.009) |
| Any adverse ALE | Intercept | 2.86 | .12 | 24.66 | .000 |
|  | Any adverse ALE | .24 | .08 | 2.91 | .004 |
|  | Time Post | -.50 | .03 | -15.11 | .000 |
|  | Time Follow-up | -.44 | .03 | -12.84 | .000 |
|  | Age | .00 | .00 | -.72 | .474 |
|  | Any adverse ALE x Time Post | -.18* | .07 | -2.51 | .012 (.031) |
|  | Any adverse ALE x Time Follow-up | -.14 | .07 | -1.87 | .062 (.109) |
| Index | Intercept | 2.84 | .12 | 24.35 | .000 |
|  | Index | .07 | .02 | 3.30 | .001 |
|  | Time Post | -.47 | .03 | -13.59 | .000 |
|  | Time Follow-up | -.42 | .04 | -11.48 | .000 |
|  | Age | .00 | .00 | -.75 | .455 |
|  | Index x Time Post | -.06*** | .02 | -3.32 | .001 (.004) |
|  | Index x Time Follow-up | -.05* | .02 | -2.64 | .008 (.022) |
| Reexperiencing (Among participants who endorsed previous ALE) (N_range_ = 170 – 259) | | | | | |
| Moderator | Parameter | B | Std.Error | t-value | p-value (BH adjusted) |
| Any ALE (N = 259) | Intercept | 2.80 | .13 | 22.25 | .000 |
|  | Any ALE | .25 | .07 | 3.39 | .001 |
|  | Time Post | -.46 | .04 | -1.89 | .000 |
|  | Time Follow-up | -.40 | .04 | -9.04 | .000 |
|  | Age | .00 | .00 | -.58 | .562 |
|  | Any ALE x Time Post | -.18* | .06 | -2.82 | .005 (.017) |
|  | Any ALE x Time Follow-up | -.19* | .07 | -2.80 | .005 (.018) |
| Any adverse ALE (N = 170) | Intercept | 2.79 | .17 | 16.33 | .000 |
|  | Any adverse ALE | .33 | .10 | 3.25 | .001 |
|  | Time Post | -.47 | .05 | -9.78 | .000 |
|  | Time Follow-up | -.41 | .05 | -8.11 | .000 |
|  | Age | .00 | .00 | -.36 | .716 |
|  | Any adverse ALE x Time Post | -.17 | .09 | -1.93 | .054 (.102) |
|  | Any adverse ALE x Time Follow-up | -.16 | .09 | -1.72 | .086 (.141) |
| Index (N = 259) | Intercept | 2.84 | .12 | 22.75 | .000 |
|  | Index | .07 | .02 | 2.96 | .003 |
|  | Time Post | -.48 | .04 | -12.42 | .000 |
|  | Time Follow-up | -.42 | .04 | -1.50 | .000 |
|  | Age | .00 | .00 | -.58 | .564 |
|  | Index x Time Post | -.05* | .02 | -2.75 | .006 (.019) |
|  | Index x Time Follow-up | -.05* | .02 | -2.41 | .016 (.040) |
| Reexperiencing (Among male participants who endorsed previous ALE) (N = 166) | | | | | |
| Moderator | Parameter | B | Std.Error | t-value | p-value (BH adjusted) |
| Any ALE | Intercept | 2.67 | .17 | 15.84 | .00 |
|  | Any ALE | .24 | .10 | 2.52 | .01 |
|  | Time Post | -.49 | .05 | -9.41 | .00 |
|  | Time Follow-up | -.41 | .05 | -7.47 | .00 |
|  | Age | .00 | .00 | .39 | .70 |
|  | Any ALE x Time Post | -.16 | .08 | -1.91 | .06 (.019) |
|  | Any ALE x Time Follow-up | -.19 | .09 | -2.15 | .03 (.040) |
| Reexperiencing (Among female participants who endorsed previous ALE) (N = 93) | | | | | |
| Moderator | Parameter | B | Std.Error | t-value | p-value (BH adjusted) |
| Any ALE | Intercept | 2.99 | .20 | 14.98 | .00 |
|  | Any ALE | .25 | .12 | 2.07 | .04 |
|  | Time Post | -.38 | .07 | -5.19 | .00 |
|  | Time Follow-up | -.37 | .07 | -4.98 | .00 |
|  | Age | -.01 | .00 | -1.44 | .15 |
|  | Any ALE x Time Post | -.24 | .10 | -2.45 | .02 (.046) |
|  | Any ALE x Time Follow-up | -.19 | .10 | -1.89 | .06 (.109) |
| *Note.* B = unstandardized coefficient; ALE = Adverse life event. B = unstandardized coefficient. BH = Benjamini and Hochberg’s (1995) False Discovery Rate adjusted p-values. N does not perfectly match sample size statistics from main text because Age contained some NA values. **p* < .05, ***p* < .01, ****p* < .005. | | | | | |

**Table S5.**

Number of participants who reexperienced not previously endorsed memory of ALE

| Types of ALE | Number of participants who reported reexperiencing an ALE that they previously did not endorse experiencing (% of full sample) |
| --- | --- |
| Any ALE | 10 (2.9) |
| Any Severe ALE | 12 (3.5) |
| Disaster | 2 (0.6) |
| Accident | 3 (0.9) |
| Physical assault | 8 (2.4) |
| Sexual assault | 9 (2.7) |
| Uncomfortable sexual experience | 17 (5.0) |
| Illness and injury | 14 (4.1) |
| Loss | 10 (2.9) |
| Perpetration | 13 (3.8) |
| Stressful experience | 29 (8.6) |
| *Note.* ALE = Adverse life event | |

**Table S6.**

Linear mixed effects model regressing Neuroticism onto the interaction between

Time and Veteran status

| Parameters | B | Std.Error | t-value | p-value |
| --- | --- | --- | --- | --- |
| Intercept | 2.84 | .04 | 80.15 | .000 |
| Sample | -.14 | .11 | -1.24 | .216 |
| Time Post | -.53 | .03 | -17.31 | .000 |
| Time Follow-up | -.46 | .03 | -14.66 | .000 |
| Sample x Time Post | -.06 | .09 | -.68 | .497 |
| Sample x Time Follow-up | -.05 | .10 | -.54 | .590 |
| *Note.* ALE = Adverse life event. B = unstandardized coefficient. N =314. | | | | |

**Table S7.**

Correlations between reexperiencing variables and baseline demographics and traits

|  | Age | Sex | Edu Level | Income | N | E | O | A | C | Trauma Healing Intention | Healing Intention |
| --- | --- | --- | --- | --- | --- | --- | --- | --- | --- | --- | --- |
| Non-veteran sample |  |  |  |  |  |  |  |  |  |  |  |
| Any adverse life event | -.06 | .24** | -.06 | -.09 | .15’ | .01 | .12 | .07 | .03 | .21** | .17' |
| Any severe adverse life event | -.10 | .25** | -.09 | -.25 | .15 | .07 | .18’ | .15 | -.02 | .25** | .10 |
| Reexperiencing Index | -.09 | .17’ | -.12 | -.16’ | .12 | .06 | .14’ | .09 | .02 | .19* | .10 |
| Veteran sample |  |  |  |  |  |  |  |  |  |  |  |
| Any adverse life event | .20 | -.17 | .08 | -.10 | .49** | -.35 | .23 | -.10 | -.31 | .27 | .17 |
| Any severe adverse life event | .17 | -.09 | .14 | .02 | .52** | -.35’ | .07 | -.15 | -.39’ | .13 | .24 |
| Reexperiencing Index | .10 | -.09 | -.08 | -.06 | .49** | -.34 | .04 | -.20 | -.34 | .22 | .16 |
| *Note.* Values indicate correlations between baseline variables and binary adverse life event reexperiencing variables.  N = Five-Factor Model Neuroticism; E = Extraversion; O = Openness; A = Agreeableness; C = Conscientiousness.  Trauma Healing Intention and Healing Intention binary variables based on qualitative coding of post-retreat open-ended responses (see Measures section for more details). | | | | | | | | | | | |
| Sample size for non-veteran sample ranges from 138 to 228; sample size for veteran sample = 32; ‘*p*<.05, **p*<.01, ***p*<.005. | | | | | | | | | | | |

**
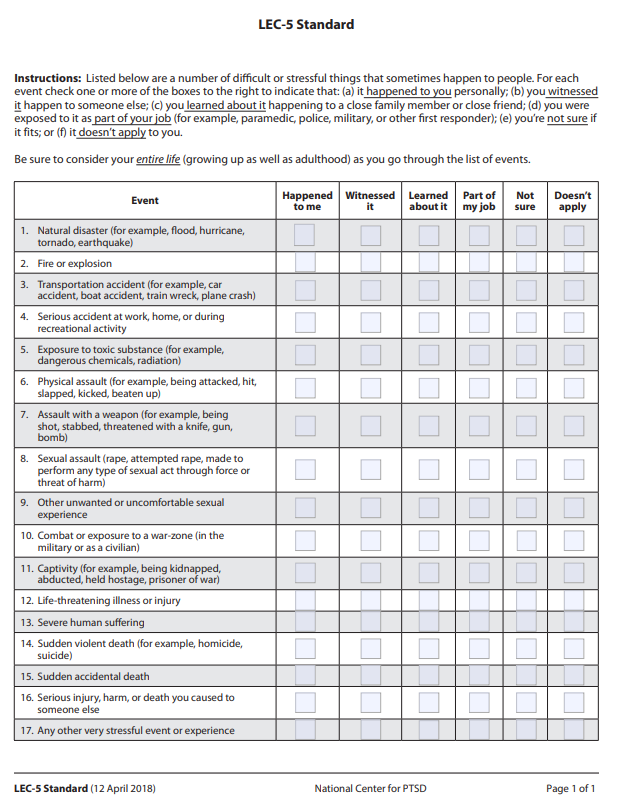
Figure S1.** LEC-5 Standard questionnaire and LEC-5 Adapted questionnaire

LEC-5 Adapted questionnaire


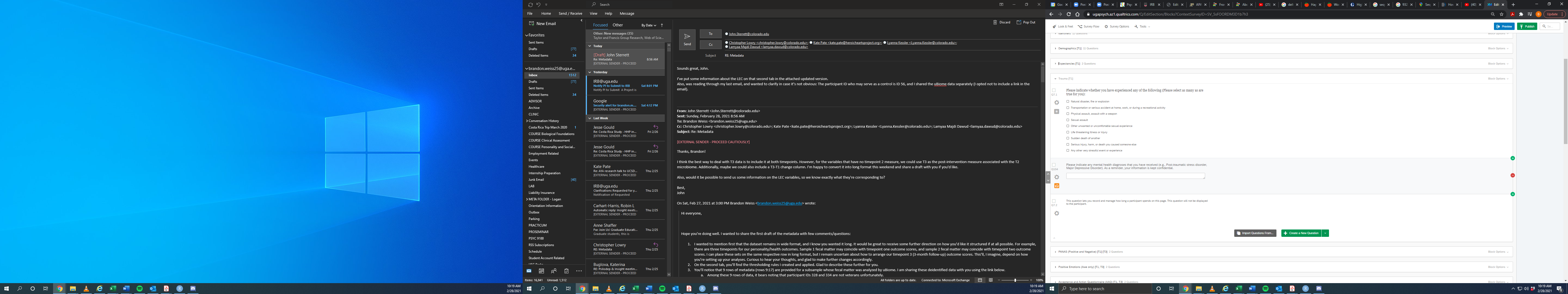


**References**

1. Weiss B, Miller J, Carter N, Campbell W. Development of the Ayahuasca Experience Inventory (AEI). Open Science Foundation (OSF). 2021.

2. Weiss B, Miller J, Carter N, Campbell W. Examining changes in personality following shamanic ceremonial use of ayahuasca. Scientific reports. 2021;11(1):1-15.

3. Agin-Liebes G, Zeifman R, Luoma JB, Garland EL, Campbell WK, Weiss B. Prospective examination of the therapeutic role of psychological flexibility and cognitive reappraisal in the ceremonial use of ayahuasca. Journal of Psychopharmacology. 2022;36(3):295-308.
